# Case Report: Diagnostic dilemma: a rare case of oesophageal hyaline vascular unicentric Castleman’s disease mimicking carcinoma

**DOI:** 10.3389/fonc.2025.1675203

**Published:** 2025-10-28

**Authors:** Qingqing Cai, Leqing Zhu, Quanwei Guo, Jun Kuang, Jianhua Zhang, Jianfeng Tan

**Affiliations:** ^1^ Department of Cardiothoracic Surgery, Shenzhen Hospital, Southern Medical University, Shenzhen, China; ^2^ Department of Thoracic Surgery, The Seventh Affiliated Hospital of Sun Yat-Sen University, Shenzhen, China

**Keywords:** Castleman’s disease, hyaline vascular type, oesophagus, diagnostic dilemma, unicentric Castleman’s disease

## Abstract

Castleman’s disease (CD) is a rare, benign lymphoproliferative disorder of unknown aetiology. CD occurring in the oesophageal region is exceedingly rare and may be misdiagnosed as oesophageal carcinoma or lymphoma, thus posing challenges for subsequent treatment selection. A 54-year-old male with a one-month history of chest pain was admitted to our hospital. Barium oesophagography and contrast-enhanced computed tomography (CT) revealed stenosis in the lower oesophagus, accompanied by wall thickening at the gastroesophageal junction. Positron emission tomography-computed tomography (PET-CT) revealed increased glucose metabolism in the oesophageal region and lymph nodes, which was suspicious for malignancy. However, a gastroscopic biopsy revealed only inflammatory granulation tissue without evidence of malignancy. Following partial oesophagectomy with intrathoracic oesophagogastric anastomosis, pathology revealed onion-skin hyperplasia of lymphoid follicles with hyalinized vessels. Combined with immunohistochemistry, these features confirmed hyaline vascular type Castleman’s disease (HV-CD). The patient exhibited good postoperative recovery. We described a rare case of oesophageal unicentric Castleman’s disease (UCD) and highlighted the significant diagnostic challenge in distinguishing oesophageal CD from oesophageal tumours preoperatively. Furthermore, we emphasized the dual significance of complete surgical resection for UCD, achieving both a definitive diagnosis and curative treatment.

## Introduction

Castleman’s disease (CD), also known as giant lymph node hyperplasia or angiofollicular lymphoid hyperplasia (1), was first described by Castleman in 1956 (2). It is a rare benign lymphoproliferative disorder of unknown aetiology (3). Clinically, CD can be classified into unicentric type (UCD) and multicentric type (MCD) (4). UCD is typically asymptomatic, whereas MCD has a poorer prognosis and is often associated with systemic symptoms such as fever, night sweats, weight loss, and anaemia. CD commonly involves lymph nodes in the mediastinum, neck, and abdomen (5). Oesophageal involvement is exceedingly rare, with only a few cases reported. Consequently, oesophageal masses are rarely considered to be CD and are often misdiagnosed as lymphoma or other tumours, which may impact subsequent clinical management decisions.

Herein, we describe a complex case of oesophageal UCD. The contradictory findings between endoscopic biopsy and imaging studies, such as positron emission tomography-computed tomography (PET-CT) and contrast-enhanced computed tomography (CT), underscore the significant diagnostic challenge in distinguishing oesophageal CD from oesophageal tumours preoperatively. Furthermore, we highlighted the dual significance of complete surgical resection for UCD, achieving both a definitive diagnosis and curative treatment.

## Case presentation

**Figure 1:**
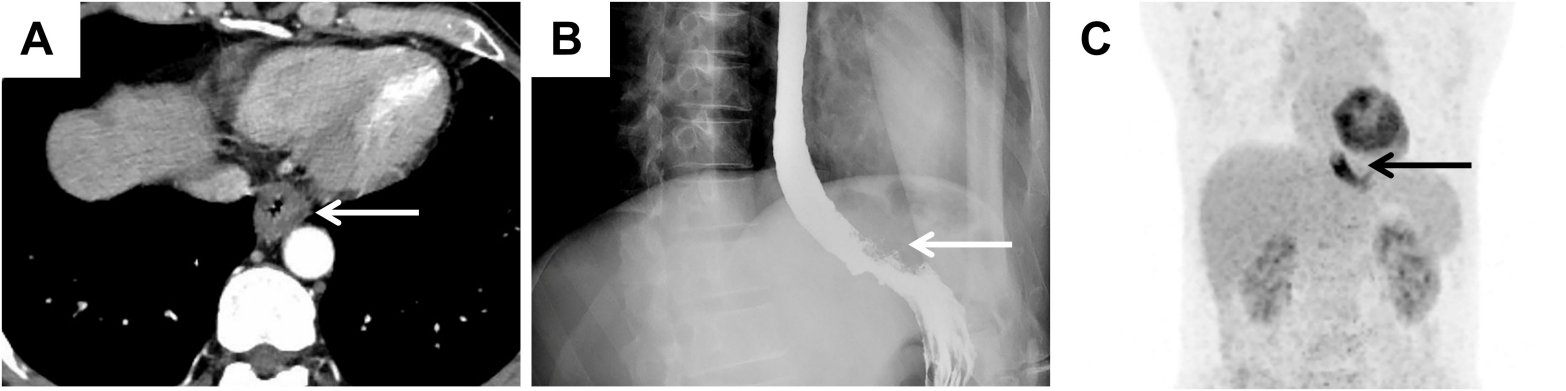
Imaging findings suggested the possibility of malignancy in the oesophagus. **(A)** CT image suggesting significant thickening of the wall of the lower oesophagus at the gastroesophageal junction (white arrow). **(B)** The results of oesophagography revealed narrowing of the distal oesophageal lumen (white arrow). **(C)** PET–CT image showing significant thickening of the wall of the lower thoracic oesophagus with increased glucose metabolism (black arrow).

A 54-year-old male presented with chest pain that had persisted for over one month. This patient did not exhibit fever or other inflammatory symptoms. The laboratory reports indicated elevated CD-related inflammatory markers, including: C-reactive protein 8.66 mg/L (normal range 0.00-5.00mg/L); erythrocyte sedimentation rate 24 mm/h (normal range < 15 mg/L); Immunoglobulin G 23.4% (normal range 9.2-18.2%); Interleukin-6 (IL-6) 7.28 pg/ml (normal range 0.00-5.30 pg/ml). Test results for the infections of human herpesvirus 8 (HHV-8) and human immunodeficiency virus (HIV) were all negative. CT revealed wall thickening at the gastroesophageal junction (**Figure 1A**). Oesophagography revealed stenosis in the lower oesophageal lumen (**Figure 1B**), whereas PET-CT revealed increased glucose metabolism at the oesophageal lesion site (**Figure 1C**). No distant metastases were detected in the left paraesophageal, bilateral cervical, bilateral supraclavicular, or axillary lymph node regions. These imaging findings suggested possible malignancy in the distal oesophagus.

**Figure 2:**
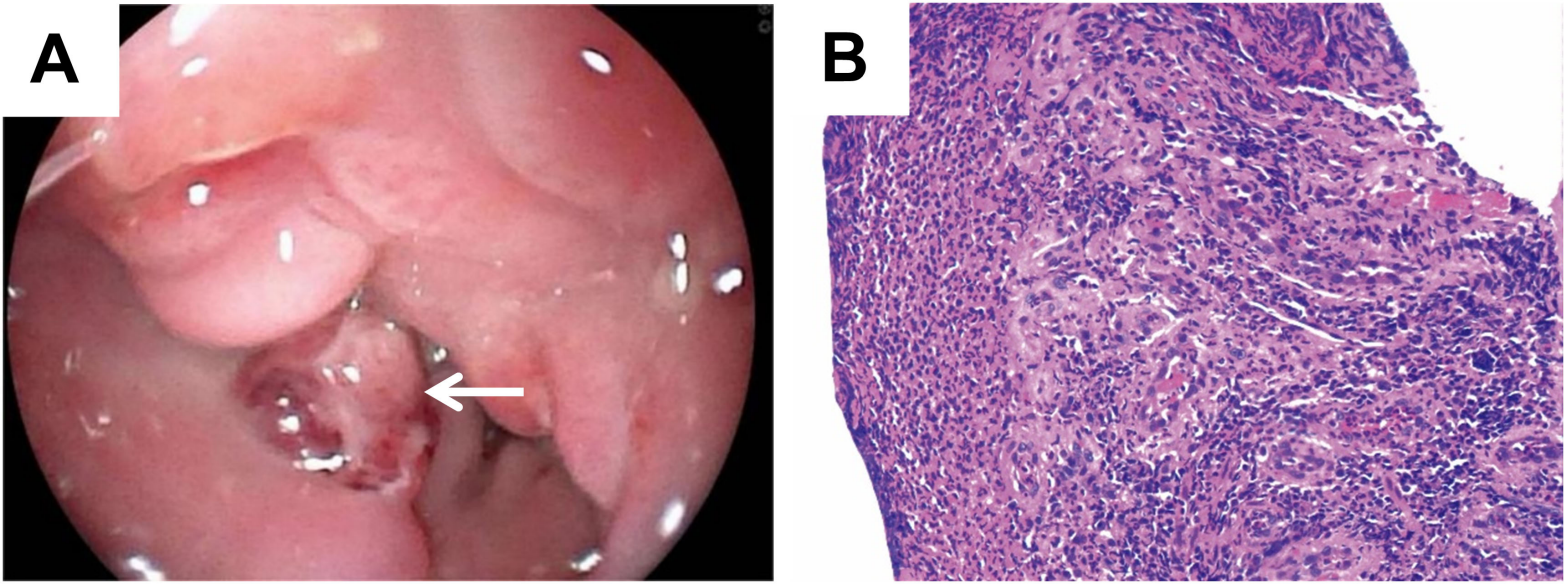
Gastroscopic examination and biopsy showed benign changes. **(A)** Gastroscopy revealed a protruding mass with luminal narrowing in the lower oesophagus (white arrow). **(B)** Pathology of gastroscopic biopsy revealed inflammatory lesions (H&E, magnification: ×10).

However, gastroscopic examination and biopsy indicated inflammatory granulation tissue at the lesion site, with no evidence of malignancy (**Figures 2A, B**). Imaging and biopsy results complicated the preoperative diagnosis. To establish a definitive diagnosis and initiate treatment, the patient underwent partial oesophagectomy with intrathoracic oesophagogastric anastomosis and additional intraoperative biopsy.

**Figure 3:**
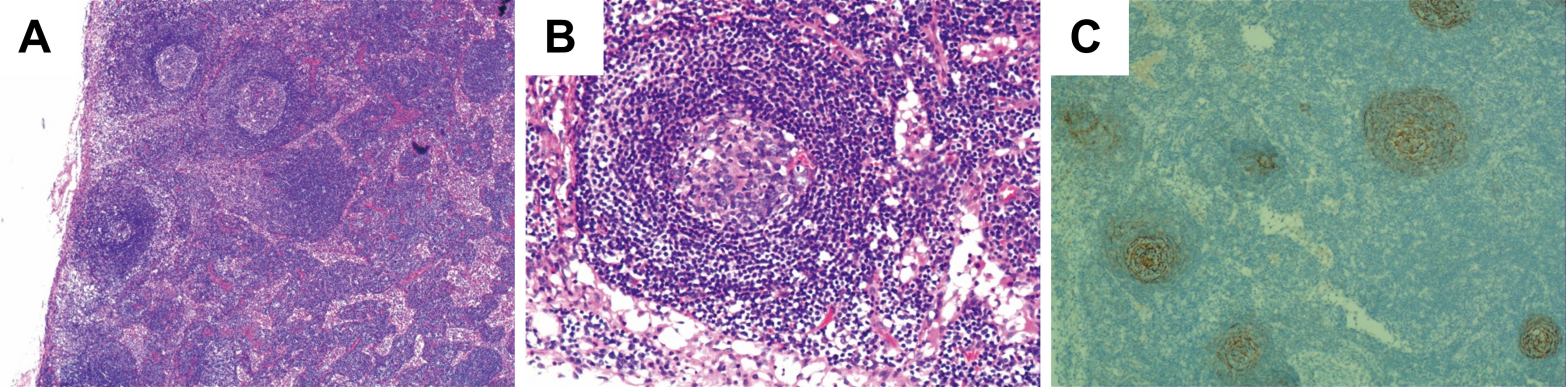
Postoperative pathologic findings demonstrated typical lymph node manifestations in CD. **(A, B)** Conventional pathological microscopic results of the postoperative biopsy. H&E, A, Magnification: 400×; B, Magnification: 400×. **(C)** Follicular dendritic cells of the germinal centres stained with CD21 antibodies.

Microscopic examination of the surgical specimens showed extensive patchy necrosis and exfoliation of the oesophageal epithelium, resulting in tissue defects. The surface was covered with inflammatory necrotic exudate and exhibited granulation tissue formation. Postoperative pathological results revealed increased lymphoid follicles, regressed germinal centres, expanded mantle zones of lymphocytes, and a proliferated interfollicular vasculature, forming an onion-skin configuration, as shown in **Figures 3A, B**. Immunohistochemical staining further demonstrated positive expression of CD20 and CD79a in B cells, whereas CD3 was positively expressed in T cells. Additionally, CD21 (**Figure 3C**) and CD23 were positively expressed in follicular dendritic cells. Ki-67 expression was significantly elevated, exceeding 20%. Based on these findings, the patient was diagnosed with hyaline vascular type Castleman’s disease (HV-CD). The postoperative course was uneventful; the patient recovered well and was discharged. A one-year follow-up examination revealed no signs of recurrence. We conducted long-term follow-up examinations to monitor the patient’s subsequent recovery and recurrence status.

## Discussion and conclusion

CD is a rare chronic lymphoproliferative disorder first reported by Benjamin Castleman in 1956 (2). Its clinical subtypes include UCD and MCD, while the histopathological subtypes are classified as HV-CD, plasma cell type (PC-CD), and mixed type. Previous studies have suggested that dysregulated expression of IL-6, activation of interferon regulatory factor 3 (IRF3), and infections with HHV-8 and HIV contribute to its pathogenesis (6). HHV8 infection is a major driver of HHV8-MCD. The virus replicates in lymph node plasma cells, contributing to systemic inflammatory symptoms and lymph node lesions, as well as a range of other cytokines, including IL-6 (7). However, the exact mechanisms remain unclear.

Currently, cases of idiopathic UCD involving the oesophageal mucosa are exceedingly rare and pose significant challenges in the differential diagnosis from early-stage oesophageal carcinoma (8), critically impacting subsequent treatment selection. This diagnostic difficulty arises from several factors. First, literature reports indicate that lymph node involvement in CD predominantly occurs in the abdomen (35. 7%), cervical (25. 7%), mediastinal (23. 1%), and axillary (6. 4%) regions (9), Some cases also occur in rare areas including the gastrointestinal tract (10) or liver (11), with oesophageal involvement documented in only a few cases (5). Consequently, these rare CD diseases, which manifest in specific regions, can be misinterpreted as malignant changes, thereby impeding clinical evaluation and the selection of subsequent treatment.

Second, the absence of specific clinical symptoms, laboratory markers, or imaging features makes histopathological examination the gold standard for definitive diagnosis, contributing to diagnostic challenges. As reported by Ki Nam Kim et al., a case occurring in the oesophageal submucosal layer further highlights that oesophageal-involved CD can easily be confused with lymphoma or other tumours (8). Furthermore, unlike previously reported cases of UCD in the oesophageal region, this patient exhibited significant wall thickening and luminal stenosis, with imaging manifestations more closely resembling carcinoma, posing a considerable diagnostic challenge. Diagnostic complexity also escalates when CD coexists with oesophageal squamous cell carcinoma (12), frequently leading to diagnostic delays or missed diagnoses.

More importantly, the patient in this case presented unique characteristics. The patient initially sought medical attention for chest pain but exhibited no typical oesophageal cancer symptoms, such as progressive dysphagia. Simultaneously, gastroscopy revealed an inflammatory mass rather than a malignant tumour. However, a preoperative PET–CT scan revealed increased glucose metabolism at the site, suggesting malignancy, which contradicts the results of the gastroscopic biopsy. The final diagnosis of UCD was confirmed only after partial oesophagectomy with intrathoracic oesophagogastric anastomosis and a second biopsy, which demonstrated classic histopathological features of HV-CD. The difference between imaging studies and biopsy findings during this diagnostic process underscores the complexity and uniqueness of this UCD case.

Regarding treatment selection for UCD, this case is consistent with the existing literature. Surgical resection remains the primary treatment for UCD (13), with most patients experiencing a favourable prognosis and low recurrence rates (14). Radiotherapy is effective for specific UCD patients (15). For MCD associated with HHV-8 or HIV infection, antiretroviral agents such as interferon (16) and chemotherapy regimens for non-Hodgkin lymphoma (17) are applicable. Recently, targeted therapies, such as siltuximab, a kind of anti-IL-6 monoclonal antibody, have been increasingly utilized in CD treatment (18). Siltuximab (11 mg/kg every 3 weeks) ± corticosteroids may serve as a first-line treatment for all patients with HHV-8-negative/idiopathic MCD cases (iMCD). The additional rounds of combination chemotherapy with or without immunomodulators/immunosuppressants are recommended if insufficient response is achieved (19). Given the localized UCD lesion in this case, complete surgical excision eliminated the need for adjuvant radiotherapy, chemotherapy, or targeted therapy. The patient recovered well during a one-year follow-up but requires ongoing monitoring due to recurrence risk (20).

In summary, this case represents a rare case of oesophageal UCD. The contradictory findings between endoscopic biopsy and imaging studies highlight diagnostic complexities, posing significant challenges in differentiating oesophageal CD from oesophageal carcinoma. We emphasized that oesophageal CD should be considered in the differential diagnosis of oesophageal space-occupying lesions, particularly when imaging and biopsy conclusions conflict. Furthermore, this case demonstrates the dual value of complete surgical resection: establishing a definitive diagnosis while achieving curative treatment in UCD (21).

## Data Availability

The original contributions presented in the study are included in the article/supplementary material. Further inquiries can be directed to the corresponding authors.
